# Microemulsion-Based Keratin–Chitosan Gel for Improvement of Skin Permeation/Retention and Activity of Curcumin

**DOI:** 10.3390/gels9070587

**Published:** 2023-07-21

**Authors:** Jiangxiu Niu, Ming Yuan, Panpan Gao, Liye Wang, Yueheng Qi, Jingjing Chen, Kaiyue Bai, Yanli Fan, Xianming Liu

**Affiliations:** College of Food and Drug, Luoyang Normal University, Luoyang 471934, China; niujiangxiu@lynu.edu (J.N.); yuanming@lynu.edu.cn (M.Y.);

**Keywords:** curcumin, microemulsion, keratin–chitosan gel, skin penetration and retention, analgesic and anti-inflammatory activity

## Abstract

Curcumin (Cur) is a kind of polyphenol with a variety of topical pharmacological properties including antioxidant, analgesic and anti-inflammatory activities. However, its low water solubility and poor skin bioavailability limit its effectiveness. In the current study, we aimed to develop microemulsion-based keratin–chitosan gel for the improvement of the topical activity of Cur. The curcumin-loaded microemulsion (CME) was formulated and then loaded into the keratin–chitosan (KCS) gel to form the CME-KCS gel. The formulated CME-KCS gel was evaluated for its characterization, in vitro release, in vitro skin permeation and in vivo activity. The results showed that the developed CME-KCS gel had an orange-yellow and gel-like appearance. The particle size and zeta potential of the CME-KCS gel were 186.45 ± 0.75 nm and 9.42 ± 0.86 mV, respectively. The CME-KCS gel showed desirable viscoelasticity, spreadability, bioadhesion and controlled drug release, which was suitable for topical application. The in vitro skin permeation and retention study showed that the CME-KCS gel had better in vitro skin penetration than the Cur solution and achieved maximum skin drug retention (3.75 ± 0.24 μg/cm^2^). In vivo experimental results confirmed that the CME-KCS gel was more effective than curcumin-loaded microemulsion (Cur-ME) in analgesic and anti-inflammatory activities. In addition, the CME-KCS gel did not cause any erythema or edema based on a mice skin irritation test. These findings indicated that the developed CME-KCS gel could improve the skin penetration and retention of Cur and could become a promising formulation for topical delivery to treat local diseases.

## 1. Introduction

Topical drug delivery refers to the application of a drug to a specific area of the skin for a local effect, rather than for systemic distribution and effect [1]. Topical drug delivery through the skin has many advantages, such as improved drug efficacy, greater patient convenience, sustained drug release, fewer times of administration, immediate dose termination when adverse effects occur and the fact that local drug concentrations are higher than systemic levels, reducing systemic side effects and providing local effects [2]. Therefore, topical drug administration is a very important and acceptable method of drug application. However, the stratum corneum of the skin is the main barrier of topically applied drugs, which limits the effective delivery of the formulation and thus leads to the reduction of local drug bioavailability [3]. Many local diseases are often found in the skin. Therefore, the maximum amount of drug penetration and retention in the skin is required to treat local diseases [4].

Curcumin (Cur) is widely used in the treatment of various local diseases such as dermatitis, acne, psoriasis, eczema, etc. [5]. The main limitation of the topical application of Cur is its poor physical and chemical properties, such as poor water solubility (13.76 μg/mL) and skin permeability, which make it difficult to achieve effective therapeutic concentration through conventional dosage forms (such as solutions, ointments or creams) [6]. Various techniques or methods have been proposed to enhance the topical delivery of lipophilic drugs of Cur [7]. One of the methods is to use nanocarriers. In recent years, many studies have been conducted on Cur-loaded nanocarriers, such as ethosomes, transfersomes, solid lipid nanoparticles, nanogels, microemulsions, niosomes, etc. [8,9,10,11,12]. Recently, the drug delivery system of nanoparticle-incorporated gel has been reported for efficient delivery of Cur [5]. The combination of nanocarriers and gel could improve the skin penetration and retention of drugs, thus improving the therapeutic effect of local diseases (such as dermatitis, eczema, pigmentation, acne, vitiligo, psoriasis, etc.) [13,14,15]. However, in the study of the combination of nanoparticles with gel formulations, a bioadhesive gel base was rarely used, and non-adhesive gel bases might have defects that could be easily cleared from the skin surface.

ME is a uniform, transparent, thermodynamically stable, isotropic colloidal carrier system composed of water, oil and emulsifier (surfactant) whose average droplet diameter is generally between 10–200 nm [16]. As a topical formulation, ME is considered a potential way to deliver drugs with poor water solubility such as Cur to the epidermis and deeper skin [17]. Actually, these ME formulations improve the skin permeability of the drug by increasing the dissolving ability of the drug. In addition, the component of surfactant in the ME formulation can reduce the interfacial tension of the skin surface, thereby reducing the diffusion barrier of the stratum corneum. Therefore, the ME formulation allows the drug to be rapidly absorbed into the skin tissue through the dense structure of the stratum corneum and increases the local accumulation of the drug [18]. However, topical application of ME to the skin is difficult because of its low viscosity and ease of removal, which results in the low residence ability of the formulation on the skin surface. Therefore, in order to improve the viscosity, spreadability and skin residence time of the ME, it is usually incorporated into a semi-solid gel matrix [19,20]. When formulating ME-based gel formulations, polymers with good biocompatibility and adhesion are the preferred gelling agents, which can extend the retention time of ME on the skin surface and reduce skin irritation.

Among the natural gelling agents, chitosan, as a natural polysaccharide, is one of the most commonly used gelling agents in drug formulations due to its unique biodegradability, biocompatibility, non-toxicity, hypoallergenicity and adhesion properties [21]. The use of natural chitosan as a gelling agent in topical ME formulations containing anti-inflammatory drugs can solve several problems, including increasing the viscosity of the ME, enhancing the epidermal adhesion of the formulations through the interaction of positively charged groups with negatively charged skin tissue, and enhancing the skin permeation/retention of the drugs by altering the keratin structure and opening tight junctions between epithelial cells [22,23,24]. Unfortunately, natural gels usually exhibit poor mechanical properties. Therefore, in order to overcome the limitations of natural gels, it is necessary to develop chitosan-based composite gels through modification and crosslinking with other materials. Recently, keratin extracted from wool, feathers or hair has become a fascinating biomaterial which shows good biocompatibility, appropriate tissue interaction and biodegradability [25]. In addition, keratin molecules have been reported to contain a variety of cell binding sites, such as LDV (Leu-Asp-Val) and RGD (Arg-Gly-Asp), which strongly support tissue adhesion [26]. Therefore, we are interested in combining chitosan and keratin by chemical conjugate to develop a topical gelling agent with excellent properties.

**Figure 1:**
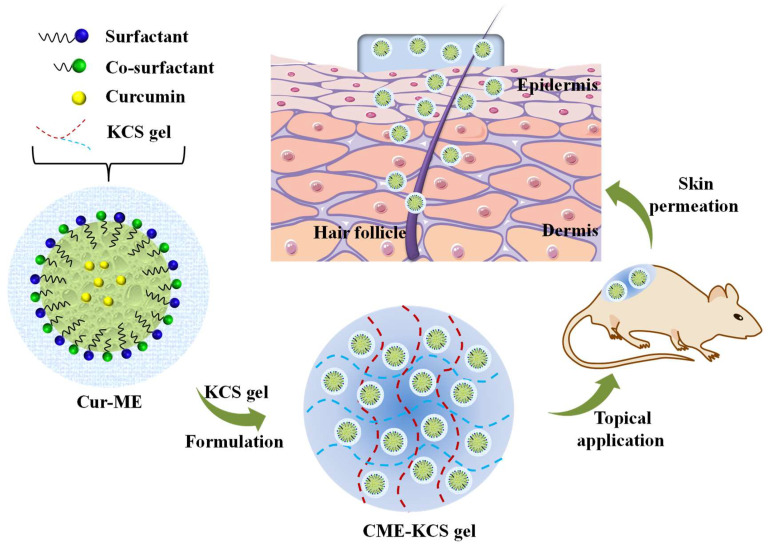
Schematic illustration of the CME-KCS gel for enhanced topical delivery of Cur.

Herein, to overcome the application obstacles of Cur in the field of topical administration, the gelling agent of a KCS conjugate was synthesized and CME-KCS gel was formulated (Figure 1). The drug-delivery characteristics and local efficacy of CME-KCS gel were evaluated in vitro and in vivo. CME-KCS gel prominently enhanced the topical delivery of Cur by taking the comprehensive advantages of the ME and KCS gels, thus producing superior local analgesic and anti-inflammatory activity. In addition, a skin irritation test confirmed that the developed CME-KCS gel formulation had good biocompatibility and safety. Therefore, this study provides a safe and promising approach to enhance the topical delivery of Cur for the treatment of local diseases.

## 2. Results and Discussion

### 2.1. Characterization of KCS Conjugate

**Figure 2:**
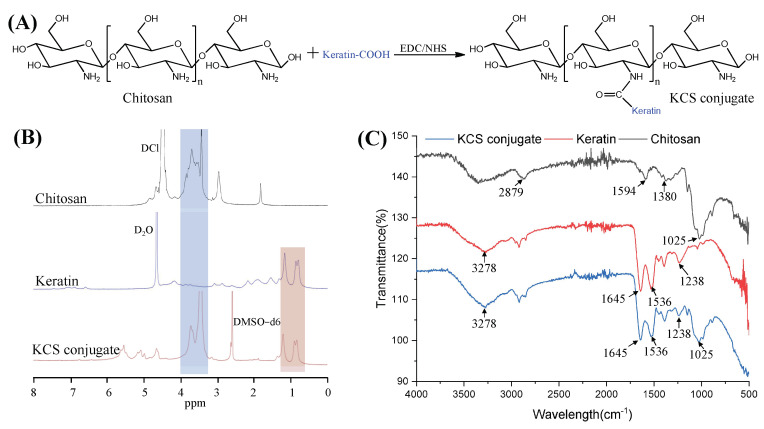
(**A**) Synthetic scheme of KCS conjugate. (**B**) ^1^H NMR spectra of chitosan, keratin and KCS conjugate. (**C**) FTIR of chitosan, keratin and KCS conjugate.

Keratin was coupled to the chitosan chain via an amide reaction between the carboxyl group of the keratin and the amino group of chitosan (Figure 2A). The KCS conjugate was confirmed by ^1^H NMR spectra (Figure 2B). The characteristic peaks, ranging from 0.76 to 1.33 ppm in the spectrum of the KCS conjugate, were attributed to protons in the keratin structure [27]. The peaks ranging from 3.14 to 3.93 ppm were attributed to protons in the sugar ring of chitosan [28]. The results confirmed that the KCS conjugate was successfully constructed. In addition, FTIR spectroscopy was also used to verify the chemical structure of the KCS conjugate (Figure 2C). In the spectrum of the keratin, the absorption peak observed at 3278 cm^−1^ was attributed to N–H stretching, and the absorption peaks observed at 1645 cm^−1^, 1536 cm^−1^ and 1238 cm^−1^ were attributed to carbonyl C=O stretching vibration (amide I), C-H stretching vibration (amide II) and C-N bending (amide III) [29], respectively. A broad band at 3600–3000 cm^−1^ was observed in the chitosan spectrum, which corresponded to the stretching vibrations of the -OH and -NH groups of chitosan, and the absorption peaks observed at 2879 cm^−1^, 1594 cm^−1^, 1380 cm^−1^ and 1025 cm^−1^ corresponded to the stretching vibration of C-H in the pyranose ring, C=O stretching vibration (amide I), N-H stretching vibration (amide II) and the stretching vibration of C-O-C in the pyranose ring [30], respectively. The spectra of the KCS conjugate contained characteristic absorptions of keratin and chitosan, indicating that the keratin and chitosan reacted successfully.

### 2.2. Characterization of Cur-ME and CME-KCS Gel

#### 2.2.1. Visual Inspection and Dyeing Test

**Figure 3:**
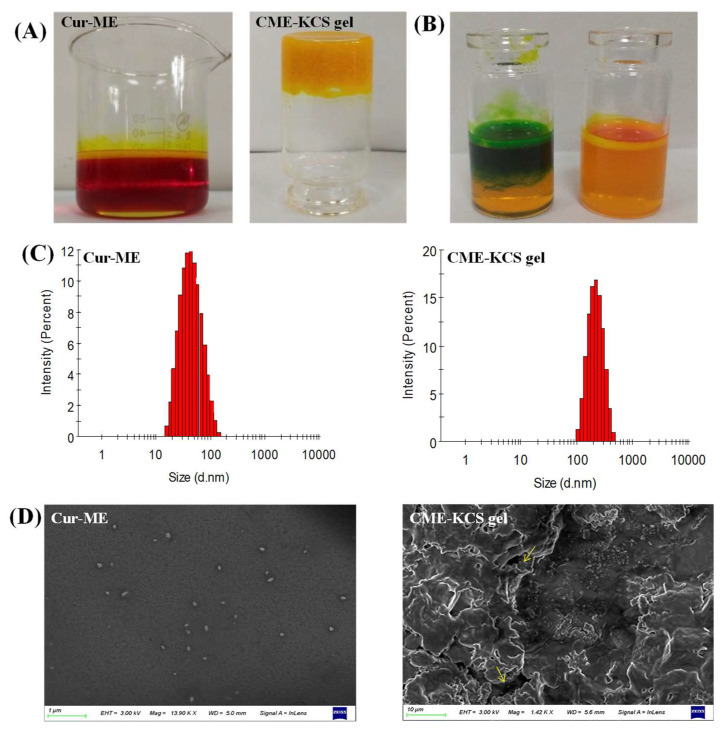
Characterization of Cur-ME and CME-KCS gel. (**A**) Appearance of Cur-ME and CME-KCS gel. (**B**) Determination of the type of Cur-ME (blue: methylene blue; red: magdala red). (**C**) Particle size distribution of Cur-ME and CME-KCS gel. (**D**) SEM image of Cur-ME and lyophilized CME-KCS gel (the yellow arrow points to the Cur-ME that was loaded into the KCS gel).

In this study, Cur was encapsulated in ME by the method of water titration. The prepared Cur-ME formed an orange-red transparent liquid, and when irradiated by laser, an obvious bright path could be observed from the vertical direction (Figure 3A), which was caused by the scattered light associated with the colloidal particles. This phenomenon is called the Tyndall phenomenon, which is the characteristic of colloidal dispersion. The developed CME-KCS gel formulation had an orange-yellow, gel-like, uniform and smooth appearance, and would not flow out when the bottle was inverted (Figure 3A). According to the characteristics of the dye used, Cur-ME was judged to be W/O or O/W type by a dyeing test. The result is shown in Figure 3B. The diffusion of the lipophilic dye magdala red was hard, while the diffusion of the hydrophilic dye methylene blue was easy in the Cur-ME preparation, so it could be inferred that the type of prepared Cur-ME belonged to the O/W style.

#### 2.2.2. Size and Zeta Potential

Cur-ME showed a particle size of 41.91 ± 0.11 nm with PDI 0.12; on the other hand, it was 186.45 ± 0.75 nm with PDI 0.27 for CME-KCS gel. The increased in particle size of the CME-KCS gel formulation could be due to the addition of the KCS gel agent, which led to the interception of oil droplets in the gel network, thus increasing the interfacial tension between the oil and water phases. A similar increase in particle size of the microemulsion after loading into a gel was reported by Nikumbh et al. [31]. Particle size plays an important role in the transdermal penetration and retention of drugs in topical applications. In general, when the particle size is less than 300 nm, the total surface area of the particle in contact with the skin is larger, and the drug has a greater chance to transfer into the skin, so it can easily penetrate the stratum corneum [32]. However, when the particle size is less than 100 nm, it is easier to penetrate into the systemic circulation through the skin, while when the particle size is more than 100 nm, it is easy to form a drug reservoir in the skin to increase the retention of drugs [33]. The particle size of CME-KCS gel is between 100 and 300 nm. Therefore, CME-KCS gel might easily cross the stratum corneum barrier and remain in the skin to form a drug reservoir, which is conducive to topical drug delivery. Cur-ME and CME-KCS gel both showed a single peak particle size distribution (Figure 3C), indicating that they were in a narrow particle size range. PDI is also a measure of particle uniformity, and the smaller the PDI value, the narrower the particle size distribution. The PDI of both Cur-ME and CME-KCS gel was less than 0.3, which further indicated that the developed formulations were mono-dispersive and not easily converted into large droplets, which would be beneficial for maintaining stability.

The zeta potential is also an important parameter for predicting particle stability. The zeta potential of Cur-ME is 0.27 ± 0.12 mV, and the near neutral potential could be explained by the non-ionic characteristics of the surfactant of pluronic F68. Due to the addition of KCS gel, the zeta potential of CME-KCS gel increased to 9.42 ± 0.86 mV. The increase in the zeta potential of CME-KCS gel might be due to the positively charged amino group in the molecular structure of chitosan, and the increase of positive charge could prevent the aggregation of droplets [34]. Therefore, the formation of the gel was beneficial to improve the stability of the preparation. In addition, the increased positive charge is more conducive to the interaction of particles with negatively charged cuticle cells, thus increasing the skin adhesion and local permeability of the preparation [35].

#### 2.2.3. SEM Imaging

The appearance of Cur-ME was spherical or spheroidal with good dispersion and uniform droplet size distribution (Figure 3D). The dimensions of the spheroids appear to be comparable to those calculated by Malvern Zetasizer. The lyophilized CME-KCS gel showed an irregular porous three-dimensional network structure (Figure 3D), which resulted from the sublimation of water during the freeze-drying process. In addition, Cur-ME could be clearly observed on the walls of the lyophilized CME-KCS gel, and it was uniformly dispersed inside the gel. The pore size of the gel network was much larger than the particle size of the Cur-ME, so these porous structures (indicated by the yellow arrows) could act as the release channels of the Cur-ME.

#### 2.2.4. pH and Viscosity

The acidic characteristics of the skin surface with pH values of 4.5 to 6.7 are considered to be an important defense mechanism [36]. The pH value of the topical formulation should be consistent with that of the skin, and changes in pH might cause irritation or damage to the skin. The pH value of the developed formulations was measured, and the results showed that the pH values of the Cur-ME and CME-KCS gel were 6.33 ± 0.01 and 6.15 ± 0.02, respectively. This is satisfactory and it was predicted that the topical application of the formulations would not cause any skin irritation.

Viscosity is considered to be one of the basic physical properties of topical formulations. The viscosity of the developed formulations was evaluated. The results showed that the viscosity of the Cur-ME and CME-KCS gel were 1.51 ± 0.245 Pa·s and 12.70 ± 0.532 Pa·s, respectively. The viscosity of the CME-KCS gel was almost eight times higher than that of the Cur-ME. The increased viscosity of the CME-KCS gel allows it to remain on the surface of the skin continuously, providing long-term action and potential targets for the treatment of local diseases [37]. Therefore, CME-KCS gel is the preferred formulation for skin drug delivery and application compared to Cur-ME.

#### 2.2.5. XRD Study

**Figure 4:**
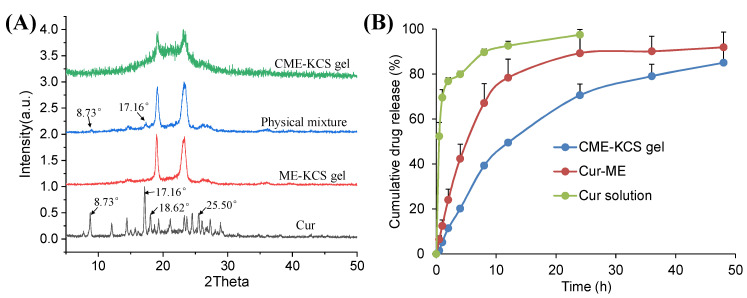
(**A**) X-ray diffraction patterns of Cur, ME-KCS gel, physical mixture and CME-KCS gel. (**B**) In vitro release profiles of Cur from the Cur solution, Cur-ME and CME-KCS gel at 37 °C (n = 3).

The XRD patterns of Cur, ME-KCS gel, the physical mixture and CME-KCS gel are shown in Figure 4A. The crystal diffraction peaks at 8.73°, 17.16°, 18.62 and 25.50° were observed in the XRD patterns of pure Cur, which indicated the crystal properties of the drug [38]. The crystal diffraction peaks of Cur at 8.73° and 17.16° were retained in the diffraction pattern of the physical mixture, indicating that Cur still exists in the crystalline form in the physical mixture. However, none of the characteristic peaks of Cur were observed in the diffraction patterns of ME-KCS gel and CME-KCS gel, suggesting that Cur existed in an amorphous form in the CME-KCS gel, and it also indicated that Cur had been encapsulated in the carrier. The amorphization of Cur offers several advantages over the crystalline form, including improved solubility and an increased rate of drug dissolution, which would be beneficial for increased drug bioavailability within the skin.

#### 2.2.6. Spreadability of CME-KCS Gel

From the point of view of patient compliance, having good spreading properties is one of the main quality characteristics of topical formulations [39]. The spreadability of blank gel and CME-KCS gel were measured. The values for blank gel and CME-KCS gel were found to be 0.27 ± 0.03 g∙cm/s and 0.93 ± 0.04 g∙cm/s, respectively. The increased spreadability of CME-KCS gel could be attributed to ME particles, indicating that the ME-based gel formulation could easily spread on the skin surface with a small amount of shear force applied [40].

#### 2.2.7. Bioadhesion of CME-KCS Gel

Bioadhesion is used to describe the adhesion ability of drug formulations to epidermal tissues [41]. The bioadhesive property of gel formulations is of key value for effective topical administration, as this property prevents the rapid clearance of the formulations from the epidermis, thereby extending the residence time of the drug in the skin and enhancing the topical activity of the drug. The bioadhesion of blank gel and CME-KCS gel were 17.29 ± 1.65 g/cm^2^ and 15.29 ± 1.28 g/cm^2^, respectively. Compared with the blank gel, the biological adhesion of CME-KCS gel was decreased, but there was no significant difference between them. The adhesion values showed that CME-KCS gel had suitable epidermal adhesion, which was attributed to the interaction between the cationic group of chitosan and the anionic group on the skin surface, and the interaction between the bioadhesive sequence of keratin and the skin surface [42,43].

### 2.3. In Vitro Release

The in vitro release curves of Cur from the Cur solution, Cur-ME and the CME-KCS gel are shown in Figure 4B. The cumulative release of the drug from the drug solution at 24 h reached 100%, while the cumulative releases of Cur-ME and CME-KCS gel at 48 h were 92% and 85%, respectively. Cur-ME and CME-KCS gel showed slow and controlled release characteristics, among which CME-KCS gel showed the most obvious performance, and its burst release performance was significantly lower than that of Cur-ME, which might be related to the fact that Cur-ME was loaded in the gel and extended the diffusion distance of the drug. In order to investigate the mechanism of drug release, drug release data were fitted into drug release kinetics to determine the best fitting model. The results showed that CME-KCS gel fitted the Korsmeyer–Peppas model (M_t_/M_∞_ = 0.1944 t^0.4262^, R^2^ = 0.9681) with the value of n less than 0.5, indicating that the release of Cur from CME-KCS gel belonged to the mechanism of Fickian diffusion. This controlled release behavior would be beneficial to play the reservoir role of the CME-KCS gel in topical application and improve the efficacy of the drug [44,45].

### 2.4. Skin Permeation and Retention

**Figure 5:**
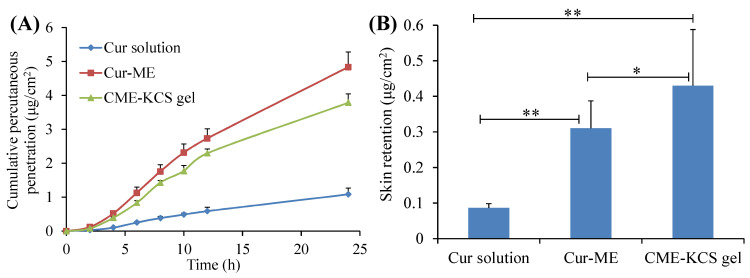
(**A**) In vitro skin permeation profiles of Cur from Cur solution, Cur-ME and CME-KCS gel. (**B**) Cur retention in rat skin after exposure to Cur solution, Cur-ME and CME-KCS gel for 24 h. Results are presented as mean ± SD (n = 3; * *p* < 0.05, ** *p* < 0.01).

The in vitro penetration curves of the Cur solution, Cur-ME and CME-KCS gel through the excised rat skin are shown in Figure 5A. It was observed that the cumulative permeability of Cur in the receiving chamber of all formulations showed a steady increase with the increase of time. At 24 h after application, the cumulative permeability of Cur from the Cur solution, Cur-ME and CME-KCS gel was 1.08 ± 0.19, 4.83 ± 0.45 and 3.78 ± 0.26 μg/cm^2^, respectively. Both the Cur-ME and CME-KCS gel showed better enhancement in Cur penetration than the Cur solution, which might be due to the fact that ingredients such as PEG400 and GTCC in ME act as permeation enhancers and could significantly change the structure of the epidermis [46]. In addition, the nanosized ME with very small droplets and a huge surface area could increase the concentration of the drug in the epidermal layer, thus causing the transfer of the drug into the skin, forming a higher concentration gradient and providing the driving force for drug penetration through the stratum corneum [47]. The cumulative permeability of Cur decreased after Cur-ME was incorporated into KCS gel to form CME-KCS gel. This might be due to the increased viscosity, which played an important role in drug diffusion into the receiving chamber. Similar results had been reported in many literatures [48,49]. Therefore, Cur diffused more rapidly in Cur-ME than in CME-KCS gel.

The skin retention of the Cur solution, Cur-ME and CME-KCS gel were 0.08 ± 0.01, 0.31 ± 0.08 and 0.43 ± 0.16 μg/cm^2^, respectively (Figure 5B). Figure 5B clearly shows that CME-KCS gel achieved maximum intradermal drug retention, which might be attributed to the great permeability-enhancing factor of the ME components, especially the nonionic surfactants and the bioadhesion of the gel [12,50]. The findings are promising because local diseases can be treated more effectively only when large amounts of the drug are retained in the skin. In addition, the side effects of the formulations used for topical treatment are mainly due to skin irritation caused by their long-term contact with the skin or the organ damage caused by the interaction between the drugs and other organs of the body after absorption into systemic circulation [40,51]. Therefore, it could be inferred from the skin retention study that the developed CME-KCS gel formulation is beneficial for improving topical bioavailability and reducing systemic adverse reactions of Cur, and has the potential to promote Cur in the treatment of local diseases.

### 2.5. Fluorescence Microscopy Imaging

**Figure 6:**
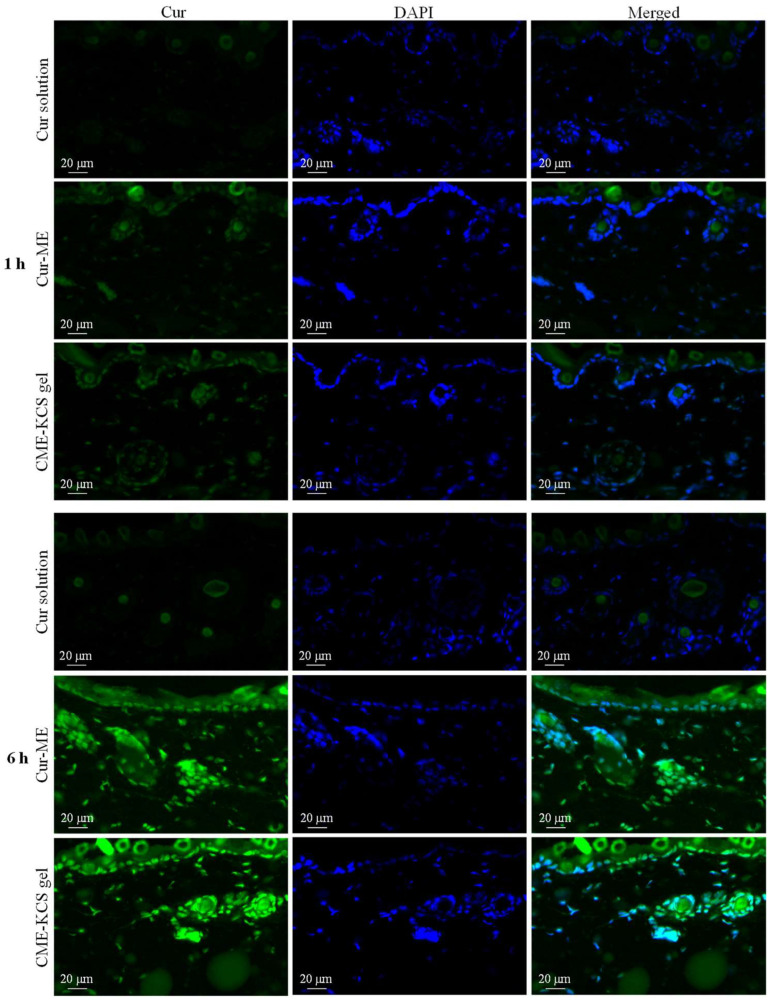
Fluorescence images of longitudinal sections of the skin incubated with Cur solution, Cur-ME and CME-KCS gel at 1 h and 6 h (the green fluorescence was derived from Cur and the blue fluorescence was derived from DAPI).

Fluorescent images of mouse skin treated with Cur solution, Cur ME and CME-KCS gel for 1 and 6 h are shown in Figure 6. At 1 h, there was almost no Cur signal observed in the skin treated with the Cur solution, which might be due to the poor permeability of the Cur molecule [6], while the skin treated with Cur-ME and CME-KCS gel showed significant green fluorescence signals of Cur in the epidermis and dermis. The fluorescence intensity of the skin treated with Cur solution, Cur-ME and CME-KCS gel significantly increased with the extension of penetration time. At 6 h, the skin treated with Cur-ME and CME-KCS gel still showed stronger Cur fluorescence when compared with the Cur solution, indicating that the developed CME-KCS gel could be used as an effective carrier to enhance the skin penetration of Cur.

It is important to note that DAPI only stains the active epidermis and dermis instead of the stratum corneum, which consists of dead cells without nuclei. According to the merged figure, the green fluorescence of the Cur appeared above the outermost edge of the DAPI stained tissue, where the stratum corneum is located (Figure 6). We inferred that the Cur-ME and CME-KCS gel could penetrate through the stratum corneum and then penetrate into the deeper skin, which might be related to the reduction of the stratum corneum barrier by the composition of Cur-ME and CME-KCS gel. In addition, we noted that hair follicles in the dermis also played an important role in transdermal delivery. It was observed that a large number of green fluorescent signals were scattered in the hair follicle; this might be due to the “giant” follicular openings of 10–210 μm in diameter [52], which make it is easier for Cur-ME and CME-KCS gel to fill hair follicles. In summary, it is inferred from the fluorescence distribution that the Cur-ME and CME-KCS gel could effectively deliver Cur to the deeper skin through the pathways of the stratum corneum and hair follicles.

### 2.6. Hematoxylin–Eosin Staining

**Figure 7:**
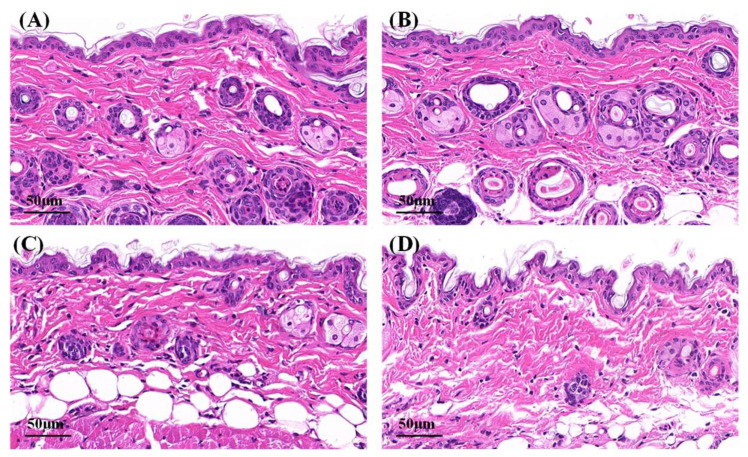
Micrographs of hematoxylin–eosin-stained mice skin sections after the mice were treated with (**A**) physiological saline, (**B**) Cur solution, (**C**) Cur-ME and (**D**) CME-KCS gel.

Microphotographs of hematoxylin–eosin-stained skin samples are shown in Figure 7. The effects of Cur solution, Cur-ME and CME-KCS gel on the mouse skin were analyzed. In the control group and Cur solution (Figure 7A,B), the stratum corneum appeared to be a complete structure with tight structures between keratinocytes. Some skin fragments could be observed on the surface of the stratum corneum, which might be caused by skin hydration after 6 h of treatment with physiological saline and Cur solution [53]. Compared with the control group and Cur solution, the stratum corneum of the mouse skin in the Cur-ME group (Figure 7C) was significantly thinner, and the keratinocytes were loose and irregular and showed larger intercellular spaces between each other. This was mainly attributed to the composition of the Cur-ME, especially PEG 400, which has the powerful function of skin penetration promotion [54]. It also explained the skin penetration mechanism of the Cur-ME. Compared with the Cur-ME, the keratinocyte space in the mouse skin was larger in the CME-KCS gel group (Figure 7D), which might be attributed to the combined action of the composition of the ME and the positive electrical groups in the chitosan structure on the stratum corneum. In general, both the Cur-ME and CME-KCS gel were effective for topical administration.

### 2.7. Assessment of Analgesic Activity

**Figure 8:**
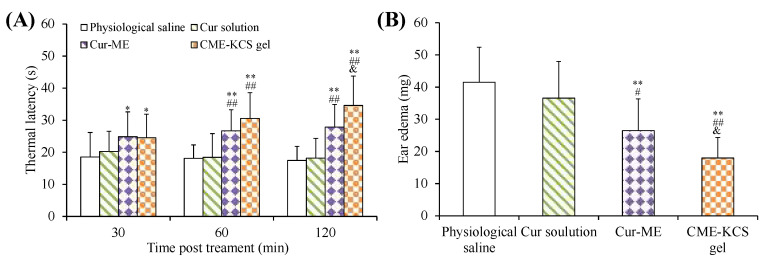
(**A**) Thermal latency of physiological saline, Cur solution, Cur-ME and CME-KCS gel in mice at various time intervals on the hot plate. (**B**) Effect of physiological saline, Cur solution, Cur-ME and CME-KCS gel on the weight of dimethyl benzene induced mice ear edema. (* *p* < 0.05 and ** *p* < 0.01 as compared to physiological saline group, ^##^ *p* < 0.01 as compared to Cur solution group, ^&^ *p* < 0.05 as compared to Cur-ME group).

The efficacy of CME-KCS gel in enhancing the topical delivery of Cur was evaluated by conducting an in vivo analgesic activity assay. The thermal latency of mice to heat stimulation pain after topical treatment with different formulations is shown in Figure 8A. As shown in Figure 8A, there was no significant change in the thermal latency of the physiological saline group and the Cur solution group during the whole 120 min of treatment. The topical application of CME-KCS gel showed significantly longer thermal latency to the thermal pain in mice at 60 min and 120 min compared with physiological saline (negative control) and Cur solution (*p* < 0.01). At 120 min of administration, the mean thermal latency of CME-KCS gel and Cur-ME was 34.63 s and 27.91 s, respectively. The thermal latency of the CME-KCS gel group was significantly extended compared with the Cur-ME group (*p* < 0.05). The permeability-promoting effect and appropriate bioadhesion of CME-KCS gel might be the main reasons for achieving higher skin bioavailability and therapeutic analgesic potential.

### 2.8. Assessment of Anti-Inflammatory Activity

The test of ear edema in mice is a suitable experimental animal model to evaluate anti-inflammatory activity. The ear edema of mice is accompanied by the release of pro-inflammatory substances, which increases capillary permeability and inflammatory cell infiltration [55]. The topical application of dimethyl benzene can cause acute skin inflammation accompanied by severe ear edema [56]. An in vivo anti-inflammatory effect was assessed in mice using the dimethyl benzene induced ear edema model. The weight difference of the right ear (treatment group) and the left ear (control group) of the same mouse was measured to monitor the severity of ear edema and evaluate the anti-inflammatory activity. The ear edema values of physiological saline, Cur solution, Cur-ME and CME-KCS gel groups were 41.51 ± 10.90, 36.58 ± 11.40, 26.46 ± 9.90 and 17.96 ± 6.39 mg (Figure 8B), respectively. Significant inhibition of ear edema was observed in the CME-KCS gel group compared with the physiological saline group (*p* < 0.01), Cur solution group (*p* < 0.01) and Cur-ME group (*p* < 0.05), which indicated that CME-KCS gel has better anti-inflammatory activity. The increase of anti-inflammatory activity of CME-KCS gel might be related to the improvement of skin penetration and retention of Cur.

### 2.9. In Vivo Skin Irritation

**Figure 9:**
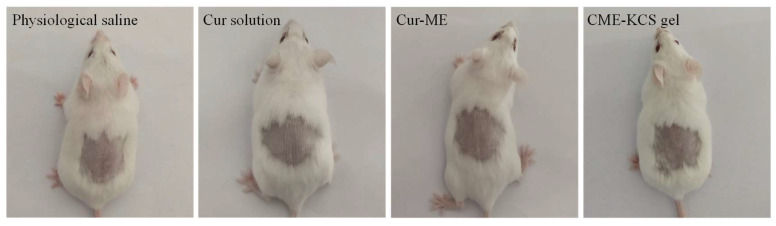
Observation of mice skin appearance at 72 h after exposure to physiological saline, Cur solution, Cur-ME or CME-KCS gel (n = 6).

Any potential irritation of the formulation might limit its use and patient acceptability. Therefore, it is necessary to evaluate the skin irritation potential of the formulation by conducting a skin irritation test. The test was performed on mice to confirm the safety and biocompatibility of the developed formulations in this study. The results of the skin irritation studies are shown in Figure 9. After 4 h of application of the Cur solution, Cur-ME and CME-KCS gel to the back skin of mice, no signs of skin erythema and/or edema were observed at 1, 4, 24, 48 and 72 h for the three test formulations. This result showed that all excipients used in CME-KCS gel were safe. Therefore, it could be considered that the developed formulations had no stimulation potential and were safe for topical application.

## 3. Conclusions

In the present study, we successfully developed CME-KCS gel for topical administration. The CME-KCS gel was characterized in terms of appearance, particle size, zeta potential, morphology, pH, viscosity, spreadability and bioadhesion. The formed CME-KCS gel had an orange-yellow and uniform appearance with a narrow particle size range and positive charge. The morphology of lyophilized CME-KCS gel showed an irregular porous three-dimensional network structure. Moreover, the CME-KCS gel exhibited desirable pH, viscosity, spreadability and bioadhesion. XRD revealed changes in Cur crystallinity, which confirmed efficient Cur loading by the CME-KCS gel. The in vitro release showed that the CME-KCS gel had slow and controlled release characteristics. In our findings, the permeation of CME-KCS gel was significantly higher compared to the Cur solution. Furthermore, drug retention in the skin of CME-KCS gel was remarkably higher when compared with the Cur solution and Cur-ME. Fluorescence imaging indicated that the CME-KCS gel could effectively deliver Cur to the deeper skin through the pathways of the stratum corneum and hair follicles. Due to the combined action of Cur-ME and KCS gel, CME-KCS gel significantly improved the analgesic and anti-inflammatory activity of Cur. In conclusion, the formulation of CME-KCS gel should be a promising formulation for topical delivery in the possible treatment of local diseases.

## 4. Materials and Methods

### 4.1. Materials

Chitosan (Mw 50 kDa, >98%) was purchased from Shanghai Maclean Biochemical Technology Co., Ltd. (Shanghai, China). (3-dimethylaminopropyl)-3-ethylcarbodiimide hydrochloride (EDC·HCL) and N-hydroxysuccinimide (NHS) were purchased from Nanjing dulai Biotechnology Co., Ltd. (Nanjing, China). Keratin was obtained from Shouhe Biotechnology Co., Ltd. (MW1500, Xi’an, China). Curcumin (≥98%) was purchased from Ivy Biotechnology Co., Ltd. (Xian, China). Polyethylene glycol 400 (PEG400) and glycerol octanoate decanoate (GTCC) were purchased from Shandong Yousuo Chemical Technology Co., Ltd. (Linyi, China). Pluronic F68 was purchased from BASF Ltd. (Shanghai, China). 4′,6-diamidino-2-phenylindole (DAPI), haematoxylin and eosin were purchased from Sigma-Aldrich (Shanghai, China). All other reagents were analytical grade.

### 4.2. Synthesis and Characterization of KCS Conjugate

The KCS conjugate was synthesized by amide reaction [57]. Briefly, chitosan (0.35 g) was dissolved in 20 mL (pH 5.0) of acetate buffer solution. Keratin (2.2 g) was dissolved in 8 mL of anhydrous dimethyl sulfoxide and stirred continuously for 1 h, and then 320 mg of EDC and 190 mg of NHS were added to the solution and reacted at room temperature for 5 h to activate the carboxyl groups of keratin. Finally, the activated keratin solution was mixed with the chitosan solution and kept at room temperature for 16 h in the dark. After the reaction, the mixture was dialyzed against deionized water with a dialysis bag (MWCO 14000) for 48 h to remove unreacted EDC and NHS, and the product obtained by freeze-drying was stored at 4 °C until use. To characterize the chemical structure of the KCS conjugate, the ^1^H NMR spectrum of the KCS conjugate was recorded using a nuclear magnetic resonance apparatus (AVANCE500, Bruker, Germany) at room temperature and the FTIR spectrum of the power of the KCS conjugate was obtained using a Fourier transform infrared spectrometer (Thermo, Fisher Nicolet iS5, Massachusetts, WA, USA).

### 4.3. Development of Cur-ME and CME-KCS Gel

Cur-ME was formulated by the method of water titration [20]. The Cur-ME formulation was prepared using pluronic F68 and PEG 400 as the surfactant and co-surfactant and GTCC as the oil. Firstly, the GTCC (5.8%) was accurately weighed into a glass vial, then the pluronic F68 (25.2%, *w*/*w*), PEG400 (50.5%) and Cur (1.0%) were accurately added to the GTCC and stirred for 10 min at 100 rpm by using Vortex equipment at ambient temperature. Subsequently, the resulting mixture was subjected to water bath sonication for 3 min and then heated to 60 °C. The mixture was stirred continuously for 15 min to obtain a homogenous solution, and then the temperature of the mixture solution was gradually reduced to room temperature with constant stirring. Finally, the deionized water (17.5%) was precisely added to the mixture drop by drop with constant stirring at 50 rpm on a magnetic stirrer until a yellow, clear and homogenous Cur-ME (equivalent to Cur 10 mg/mL) was formed. The resulting Cur-ME was stored at room temperature for 24 h to achieve equilibrium before further investigation. The KCS conjugate (20%, *w*/*v*) was added to deionized water with constant stirring to achieve complete, uniform swelling and obtain a gel base. The previously formulated Cur-ME was slowly mixed with the KCS gel base in a 1:1 ratio with constant stirring to obtain uniform CME-KCS gel, and the final formulation of CME-KCS gel contained 0.5% (*w/w*) Cur. Microemulsion-based KCS gel (ME-KCS gel) without Cur was prepared by the same method.

### 4.4. Characterization of Cur-ME and CME-KCS Gel

#### 4.4.1. Visual Inspection and Dyeing Test

The formulated Cur-ME and CME-KCS gel were visually inspected for appearance, color, uniformity and the Tyndall phenomenon. The type of Cur-ME was identified by the common staining method. Cur-ME was placed in two small glass bottles, and two different kinds of dyes, such as magdala red (lipophilic dye) and methylene blue (hydrophilic dye), were respectively added. The type of Cur-ME was identified according to the diffusion of the dye in the microemulsions system.

#### 4.4.2. Measurement of Size and Zeta Potential

Particle size and zeta potential of the Cur-ME and CME-KCS gel were determined by dynamic light scattering (DLS) using the instrument of Malvern Zetasizer (Zetasizer ZS90, Malvern Instruments, Worcestershire, UK). The Cur-ME and CME-KCS gel were respectively diluted 50 times with deionized water before measurement to ensure that the diluted formulation was within the sensitivity range of the instrument. All samples were measured at (25 ± 1) °C and performed in triplicate.

#### 4.4.3. Scanning Electron Microscope (SEM) Imaging

The morphology and external surfaces of the formulations of Cur-ME and CME-KCS gel were observed by field emission scanning electron microscope (SEM) (Sigma 500, ZEISS, Germany). In order to facilitate the observation of Cur-ME, the Cur-ME dispersion was diluted 100 times and dropped onto a silicon substrate and then placed at room temperature until completely dry. The CME-KCS gel was lyophilized at −50 °C using a vacuum freeze dryer (SCIENTZ-18N, Xinzhi, China) and then placed on double-sided conductive adhesive. All samples were gold plated in a sputter coater under vacuum prior to observation.

#### 4.4.4. Determination of pH and Viscosity

The pH of the formulations of Cur-ME and CME-KCS gel was determined at (25 ± 1) °C using a calibrated pH meter (PHS-3C, Rayci, China). In addition, viscosity is also one of the key indicators for evaluating topical transdermal formulations. The viscosity of the formulations of Cur-ME and CME-KCS gel was also measured using a rotational viscometer (NDJ-9s, Fangrui, China) to understand its adhesion on the skin surface. Spindle No. 4 was used, the temperature was set to 25 °C, and the viscosity was measured three times at a shear rate of 60 rpm/min to obtain an average value at the corresponding temperature.

#### 4.4.5. X-ray Diffraction (XRD)

To study the existing state of Cur in CME-KCS gel, the crystal structure of Cur, ME-KCS gel, the physical mixture and CME-KCS gel were analyzed by X-ray diffractometer (D8 ADVANCE, Bruker, Germany), using Cu-Kα as the source of radiation, and run at a current of 25 mA, an accelerating voltage of 40 kV and a scanning rate of 4°/min. The diffraction pattern was recorded in the 2θ scan range of 5°–50°.

#### 4.4.6. Spreadability Test of CME-KCS Gel

The spreadability of CME-KCS gel was determined by the glass slide method [58]. For this purpose, two glass slides with the same size were taken. One of the frosted glass slides (7.5 × 2.5 cm) was mounted on a wooden board. The CME-KCS gel (0.2 g) was placed on the frosted glass slide, and then another glass slide was placed over the frosted one so that the CME-KCS gel was sandwiched between two glass slides. A load of 200 g was kept on the upper slide for 3 min to expel air and provide a uniform film of the CME-KCS gel. Spreadability was expressed as the time (in seconds) required for two slides to slide off the gel placed between them with a specific load applied. It is calculated using the following formula [59]:

S = M × L/T

where S is the spreadability of the gel formulation, M is the weight associated with the slide on top, L is the length of the slide, and T is the time required to separate the slide.

#### 4.4.7. Bioadhesion Study of CME-KCS Gel

The bioadhesion of CME-KCS gel was studied in vitro. The freshly shaved mouse skin was cut into pieces with an area of 2.0 cm^2^ and washed with physiological saline. One piece of mouse skin was stuck under the right tray of the balance by double-sided adhesive with the skin surface facing down, and the other piece was attached to the surface of the test bench with the skin surface facing up. About 0.2 g of CME-KCS gel was placed between the two pieces of hairless mouse skin, the two mouse skin pieces were pressed tightly to remove the air entrained, and then water was slowly added on the left tray of the balance until the two mouse skin pieces were detached. The weight (g) of water required to detach the CME-KCS gel from the skin surfaces was noted as the force of detachment. The bioadhesive strength was calculated from the following equation [31]:

Bioadhesive strength = Weight required (g)/Area(cm^2^)

### 4.5. In Vitro Drug Release Study

The in vitro release behavior of Cur from the Cur solution (Cur dissolved in propylene glycol 400), Cur-ME and CME-KCS gel was studied by the widely used dialysis method [60]. After pre-hydration of the dialysis bag (regenerated cellulose, MWCO 3500) overnight, samples containing 1.0 mg of Cur were respectively transferred to the dialysis bag and then immersed in 40 mL of in vitro release medium (physiological saline containing 1.0% Tween 80) that completely covered the sample. The release medium was kept in a water bath at the temperature of 37 ± 0.5 °C and magnetically stirred at a speed of 300 rpm. The release medium (2.0 mL) was taken out at fixed time intervals (0.5, 1, 2, 4, 6, 8, 16, 24, 36 and 48 h), and the same volume of fresh release medium preheated to 37 °C was then immediately supplemented to maintain a constant volume. The Cur content in the samples was analyzed at 425 nm by UV-Vis spectroscopy (TU-1810PC, Purkinje, Beijing, China). Release studies were performed in triplicate for each sample. In order to study the drug release mechanism, the release data were fitted to zero-order, first-order, Higuchi diffusion and Koresmeyer–Peppas kinetic models, and the correlation coefficient was used as the index of goodness of fit [61,62].

### 4.6. In Vitro Skin Permeation and Retention Study

#### 4.6.1. Preparation of Rat Skin

The abdominal skin of female SD rats (180–220 g) was used for the skin penetration and retention study. The rats were sacrificed by inhalation anesthesia with ether and the hair was carefully removed with an electric shaver. The depilated skin of rats was excised, and fat and adhesive tissues were removed. The skin was rinsed with physiological saline and stored at 4 °C with the restriction of usage within 24 h. Skin integrity was checked visually before use.

#### 4.6.2. In Vitro Skin Permeation Study

The Franz diffusion cell with an effective diffusion area of 2.8 cm^2^ and a volume of 6.5 mL in the receiving chamber was used for the skin penetration study. The receiving chamber was filled with 6.5 mL of physiological saline containing 1.0% Tween 80 as the receiving solution. The temperature was maintained at 32 ± 2 °C using a circulating water bath and magnetically stirred at a constant speed of 300 rpm. The skin membrane was cut into appropriate size and mounted between the donor and the receiving chamber with the epidermis facing the donor chamber and the dermis facing the receiving solution. The Cur solution, Cur-ME and CME-KCS gel were placed on the epidermis of the donor chamber. Samples of 2.0 mL were taken at predetermined time points (2, 4, 6, 8, 10, 12, 24 h) and then immediately supplemented with an equal volume of fresh receiving solution to maintain a constant volume. After filtering the samples with a microporous membrane (0.22 μm), the content of Cur was determined by HPLC and the cumulative transdermal permeation amount was calculated. The HPLC system (U-3000, Thermo, Waltham, MA, USA) applied included a reversed-phase WondaSil C18 column (5 μm, 250 × 4.6 mm) and UV detector operated at the wavelength of 423 nm. The mobile phase was composed of acetonitrile and 0.5% phosphoric acid (58:42, *v*/*v*) that was filtered with organic membrane (0.22 μm), degassed by ultrasound and delivered at a flow rate of 1.0 mL/min. The temperature of the column chamber was maintained at 30 °C, and the injection volume was 20 μL.

#### 4.6.3. Skin Retention Study

After completion of the skin permeability experiment, the skin sample was removed from the Franz diffusion cell, the residual samples on the skin surface were cleaned with deionized water three times and then were wiped with alcohol cotton balls three times. The skin was drained by filter paper, cut into pieces and immersed in 1.0 mL of methanol at 4 °C for 24 h. Subsequently, the ultrasonic treatment was conducted for 1.5 h to fully extract the Cur remaining in the skin. The retention of Cur in the skin was determined by HPLC.

### 4.7. Fluorescence Imaging

As Cur has natural fluorescence properties, its skin penetration depth and intradermal distribution can be observed by fluorescence imaging. The back hair of mice was removed 12 h before the experiment. After the administration of the Cur solution, Cur-ME and CME-KCS gel to the hair removal area for 1 h and 6 h, the mice were sacrificed by excessive inhalation of ether. The treated skin was excised, rinsed with physiological saline and dried with cotton swabs, and the skin samples were frozen at −20 °C with freezing solution. The frozen samples were cut vertically into 8 μm sections with a cryomicrotome (Leica CM 1950, Wetzlar, Germany), the sections were stained with DAPI and scanned with a digital scanner (3DHISTECH, Ltd., Budapest, Hungary) and the fluorescence images were viewed to understand the skin penetration depth and localization of Cur.

### 4.8. Hematoxylin–Eosin Staining

Hematoxylin–eosin staining was carried out to observe the histology of the excised skin. After the backs of the mice were shaven, Cur solution, Cur-ME and CME-KCS gel were administered locally for 6 h, and then the mice were killed by inhaling excessive ether, and the skin of the mice was removed. The skin was fixed with 4% formaldehyde, embedded in paraffin, and vertical sections with a thickness of 5 μm were made using a microtome (LEICA RM2235, Nussloch, Germany). After the skin sections were stained with hematoxylin and eosin, the digital slide scanner (3DHITECH, Ltd., Budapest, Hungary) was used to obtain the image of the skin sections. The matching analysis software of CaseViewer 2.3 was used to analyze the influence of the different formulation on the skin microstructure.

### 4.9. Analgesic Activity Evaluation

The in vivo analgesic activity was evaluated by a hot plate test. The hot plate test was performed using a thermostatic smart hot plate instrument (YLS-6B, Jinan, China) maintained at 55 ± 1 °C. The reaction time(s) or latency was determined as the time required for mice to respond to heat pain by licking their paws, retracting their paws or jumping. Mice with basal latency of 5–30 s were selected for analgesic activity evaluation. Physiological saline was used as a negative control, and Cur solution, Cur-ME and CME-KCS gel were applied topically to the plantar of the mice respectively for 30 min at a dose of 15 mg/kg. The reaction time was recorded 30, 60 and 120 min after treatment administration. The maximum reaction time was fixed at 60 s to prevent damage to mouse paw tissue. If the mice did not respond to the heat pain at 60 s, 60 s was considered the maximum analgesia.

### 4.10. Anti-Inflammatory Activity Evaluation

The mice were randomly divided into 5 groups with 10 mice in each group. Physiological saline (negative control), Cur solution, Cur-ME and CME-KCS gel were evenly applied to both sides of the right ear, and the left ear was used as a blank control without any treatment. After treatment for 1 h, dimethyl benzene (30 μL) was locally applied to both sides of the right ear to induce ear edema in mice. After inducing ear edema for 30 min, the mice were sacrificed by excessive inhalation of ether, the ears were carefully cut with a puncher (8 mm in diameter) and the weight difference between the left and right ears was measured to evaluate the therapeutic effect.

### 4.11. Skin Irritation Test

A skin irritation test was carried out using mice as subjects. The skin of the back area was shaven using an electric hair remover 24 h before the experiment. Physiological saline (control group), Cur solution, Cur-ME and CME-KCS gel were respectively applied to the depilated area (approximately 1.5 cm × 1.5 cm) of six mice for 4 h, and the remaining test sample was removed with warm water. Each application site was observed and recorded at 1, 4, 24, 48 and 72 h after removal of the test sample and then assessed for any adverse signs and symptoms such as erythema and/or edema.

### 4.12. Statistical Analysis

All experimental data were expressed as mean ± standard deviation (SD). At least three independent experiments were performed for each experiment to obtain statistical data. The significance of the comparison data between the two groups was analyzed by independent *t*-test and statistical differences were determined, and a *p* value < 0.05 was considered to be statistically significant.

## Data Availability

The raw/processed data required to reproduce these findings cannot be shared at this time as the data also form part of an ongoing study.
